# The challenge of neuropsychological assessment of visual/visuo-spatial memory: A critical, historical review, and lessons for the present and future

**DOI:** 10.3389/fpsyg.2022.962025

**Published:** 2022-08-23

**Authors:** Unai Diaz-Orueta, Bronagh M. Rogers, Alberto Blanco-Campal, Teresa Burke

**Affiliations:** ^1^Department of Psychology, Maynooth University, Maynooth, Ireland; ^2^Department of Psychology, Faculty of Arts, Humanities and Social Sciences, University of Limerick, Limerick, Ireland; ^3^Department of Psychiatry for the Older Person and Memory Clinic Services, Health Service Executive, Ardee and Navan, Ireland; ^4^Faculty of Science and Health, School of Psychology, Dublin City University, Dublin, Ireland

**Keywords:** nonverbal memory, visual memory, spatial memory, neuropsychological assessment, virtual reality

## Abstract

A proliferation of tests exists for the assessment of auditory-verbal memory processes. However, from a clinical practice perspective, the situation is less clear when it comes to the ready availability of reliable and valid tests for the evaluation of visual/visuo-spatial memory processes. While, at face value, there appear to be a wide range of available tests of visual/visuo-spatial memory, utilizing different types of materials and assessment strategies, a number of criticisms have been, and arguably should be, leveled at the majority of these tests. The criticisms that have been directed toward what are typically considered to be visual/visuo-spatial memory tests, such as (1) the potential for verbal mediation, (2) over-abstraction of stimuli, (3) the requirement of a drawing response, and (4) the lack of sensitivity to unilateral brain lesions, mean that, in reality, the number of readily available valid tests of visual/visuo-spatial memory is, at best, limited. This article offers a critical, historical review on the existing measures and resources for the neuropsychological assessment of visual/visuo-spatial memory, and it showcases some examples of newer tests that have aimed to overcome the challenges of assessing these important aspects of memory. The article also identifies new trends and examples of how technological advances such as virtual reality may add value to overcome previous obstacles to assessment, thereby offering professionals more reliable, accurate means to evaluate visual/visuo-spatial memory in clinical practice.

## Introduction

Memory refers to the complex processes by which the individual encodes, stores, and retrieves information (Strauss et al., 2006). As Lezak et al. (2012) state, many common neurological and psychiatric conditions produce a decline in the efficiency of memory processes, so that memory assessment often becomes the central issue in a neuropsychological examination. In this context, although the accepted view of memory is of a multi-faceted construct (e.g., Atkinson and Shiffrin, 1968; Tulving, 1972, 1985; Mayes, 1992), thorough examination of memory, in all its facets, is rarely, if ever, undertaken in clinical practice. Rather, with relatively few exceptions, the focus is typically on evaluation of explicit anterograde episodic memory, hereon referred to as episodic memory.

For almost 50 years now, hemispheric differences in episodic memory have been proposed, with verbal memory, whatever the mode of stimulus presentation, considered to be mediated by the left hemisphere and nonverbal memory (typically nonverbal visual) considered to be mediated by the right (Milner, 1968a, 1970; Jones-Gotman, 1986; Barr, 1997; Strauss et al., 2006; Baxendale, 2008; Willment and Golby, 2013), at least in those left-hemisphere dominant for language. It is useful, therefore, theoretically and clinically, to distinguish between episodic memory tests on the basis of the type of material they employ (e.g., verbal vs. nonverbal; a *material-specific* categorization) and not just on the basis of the sensory modality employed to present and/or to process the material (e.g., auditory, visual or tactile; a *modality specific* categorization).

For the purpose of operational definition, “Material-specific memory refers to the ability to learn and recall new episodic information on the basis of the nature of the stimulus material (e.g., verbal vs. nonverbal-visuospatial). Modality-specific memory refers to the ability to learn and recall new episodic information on the basis of the sensory modality of stimulus presentation (e.g., auditory vs. visual)” (Vanderploeg et al., 2001, pg. 174).

Evidence of material-specific memory deficits that are dependent upon laterality of lesion has, as Strauss et al. (2006) so eloquently pointed out, been somewhat elusive in clinical practice at least as far as nonverbal memory deficits associated with right-hemisphere lesions are concerned. They went on to say: “… it is unclear whether difficulties in documenting the existence of material specific impairments in nonverbal memory are the result of problems with the test procedures or of lack of validity of the entire construct of nonverbal memory (Barr et al., 2004)” (Strauss et al., 2006, pg. 680).

In many clinical contexts, inclusion of both verbal and nonverbal memory tests appears warranted, particularly when issues of laterality of lesion are at issue, but appropriate test selection is often not a simple task. Indeed, test selection is made difficult by the paucity of readily available tests of (truly) nonverbal memory. The purported nonverbal memory tests that are readily available for clinical practice rely on the use of visual stimuli rather than on other types of nonverbal material (such as nonverbal sounds). Thus, the emphasis in this paper is on the critical evaluation of widely used visual memory tests that have, to a greater or lesser degree, been characterized as representing tests of nonverbal episodic memory.

In order to provide some clarity on the current status and trends of neuropsychological assessment of nonverbal episodic memory, the goal of this paper will be first to identify challenges in assessing visual and visuospatial memory, and then to offer a critical, historical review on existing measures and resources for the neuropsychological assessment of visual and visuospatial memory, and to showcase some examples of newer tests and alternative methodologies that have aimed to overcome the challenges of assessing this important aspect of episodic memory. This is not intended as a systematic review, but rather a broad overview of assessment tools widely used in clinical practice. A third goal of the paper will be to identify a number of new trends and examples of how technological advances, such as virtual reality, may add value to overcome previous obstacles to assessment, thereby offering professionals more reliable, accurate means to evaluate visual/visuo-spatial episodic memory in clinical practice.

## Neuropsychological assessment of visual memory: A historical overview of the challenges

A proliferation of assessment tools exists for the assessment of specific aspects of verbal memory (see, for example, Lezak et al., 2012; Sherman et al., 2022). However, from a clinical practice perspective, the situation is less clear when it comes to the ready availability of reliable and valid assessment tools for the evaluation of nonverbal memory. As noted by Wechsler (2009a), “the creation of a ‘pure’ visual memory task is very challenging, as many methodological and construct-related issues unrelated to memory functioning confound the interpretation of these tests (Heilbronner, 1992).” Moreover, the tendency to verbalize memories when sharing our life events (for example, a memory of a conversation or a memory of a walk on a beach) renders things difficult when trying to “purely” separate the verbal aspect of episodic memory from the “visual/visuo-spatial” aspect and *vice-versa*.

The main problem for the assessment of visual memory abilities, whether or not one is attempting to assess nonverbal visual memory, relates to how examination is currently undertaken in clinical practice. One of the most persistent challenges in the evaluation of nonverbal memory relates to the difficulties encountered historically in the development of a “pure” measure of nonverbal visual memory (the most frequent approach used in attempts to assess nonverbal memory function) without the confound of significant verbal mediation or the impact of other cognitive processes that may either mediate or interfere with visual memory performance. The latter criticism is, of course, not unique to nonverbal tests but also applicable to most cognitive instruments used in clinical practice, which require multiple cognitive abilities for successful performance and, therefore, unravelling the dominant neurocognitive mechanism of the impairment can be challenging (Kaplan, 1988).

Typically, available measures require the test taker to recognize previously presented visual designs of varying complexity (e.g., Recurrent Figures Test—Kimura, 1963, Continuous Recognition Memory Test—Hannay et al., 1979, and Continuous Visual Memory Test—Trahan and Larrabee, 1988) or unfamiliar faces in recognition memory paradigms such as in the Warrington Recognition Memory Test-Faces subtest (Warrington, 1984) or the Wechsler Memory Scale Third Edition Faces subtest (WMS-III: Wechsler, 1997a,b) or to draw and later recall geometric designs (e.g., Visual Reproduction subtests from the WMS Scales Wechsler, 1997a,b, 2009b), the Rey Complex Figure Test (Meyers and Meyers, 1996), its alternate, the Taylor Figure (Taylor, 1969, 1979); or its modified version (Hubley and Tremblay, 2002) or the more recent four Medical College of Georgia complex figures (Loring and Meador, 2003). Others focus on assessment of memory for spatial location (7/24 Test: Rao et al., 1984) to the exclusion of memory for visual detail. Remarkably few assess memory for both visual details of the test stimuli and memory for location and even then, performance on these two components of visuospatial memory are typically combined in scoring rather than treated as distinct components of a complex process that are likely mediated by distinct neuroanatomical regions. By way of example, visual recognition memory is considered to be mediated by lateral temporal regions of the right hemisphere (Milner, 1968a; Burke and Nolan, 1988; Burke and Milner, 1991), while memory for location is mediated by the hippocampus and associated regions, with noted involvement of both left and right mesial temporal structures (see Smith and Milner, 1981, 1989; Maguire, 1994; Maguire et al., 1996).

As can be inferred, a range of cognitive processes, other than those related directly to visual memory are involved in successful completion of these types of tasks. Any test designed to evaluate visual memory that requires a visuomotor response (e.g., drawing or construction) will draw on processes related to complex perceptual analysis and constructional abilities; interaction between memory and constructional processes; and even potential hemispatial inattention related problems that may confound the clinical picture even further (Lezak et al., 2012). Unless a process-based approach to neuropsychological assessment is employed, clinical interpretation may well be flawed (Kaplan, 1988; Libon et al., 2013). If the assessment of visual memory includes a spatial component in what we may call assessment of visuospatial memory, it is important to recognize that such tests may also assess other areas of functioning, such as executive functioning and sensorimotor abilities (see for example, Edidin and Hunter, 2013), complicating clinical interpretation further still.

Thus, while, at face value, there appear to be a wide range of available tests of visual memory, utilizing different types of materials and assessment strategies, a number of criticisms have been, and arguably should be, levelled at the majority of these tests—particularly if they are to be characterized as nonverbal memory tests. The criticisms that have been directed toward ostensibly nonverbal or visual memory tests that will be detailed in this paper can be summarized as follows: (1) the potential for verbal mediation, (2) over-abstraction of stimuli, (3) the requirement of a drawing response or some kind of motor performance (closely linked to perceptual function, attention, memory and spatial orientation—Beaumont and Davidoff, 2019), and (4) the lack of sensitivity to unilateral brain lesions; all of which mean that, in reality, the number of readily available valid and meaningful tests of nonverbal visual memory is very limited. This point will be discussed further in reference to our overview of clinical assessment measures. Meanwhile, a brief look at each of these issues follows.

### The problem of verbal mediation

The problem of verbal mediation arises because of the nature of the task. Here, the stimuli presented to the patient and/or the task that the patient is asked to perform, permits the individual to rely, to a greater or lesser degree, on verbal mnemonics in order to demonstrate evidence of memory for visual stimuli. An exemplar of this type of verbal mediation is seen in a wide range of visual reproduction tasks where the test taker is asked to recall a simple geometric design or designs that often lends themselves to verbal labelling (e.g., Benton Visual Retention Test, Benton, 1974; Sivan, 1992). Another is the Family Pictures (FP) subtest of the Wechsler Memory Scale-Third Edition (Wechsler, 1997a). The task, as described by Dulay et al. (2002), involves the initial presentation of a “family portrait” of six family members and their dog. The examiner identifies the characters and tells the examinee that they are the characters that will appear in four subsequent scenes (e.g., an older adult sitting on a bench while the dog is playing with a frisbee). Each scene is exposed for 10 seconds while the examinee is told to remember as much as possible about the scene. After all four scenes have been viewed, the examinee is asked to recall information about each scene, including who was in the scene (characters), where the characters were located (location), and what each character was doing (activity). In the delayed testing phase (FP II), the examinee is again asked to recall scene characters, spatial location, and activities. However, this subtest does not actually assess the individual’s memory for the visual detail of the stimuli contained within the scene. In other words, the testee is not required to demonstrate, through forced-choice recognition, or otherwise, that they recollect what any particular individual seen in the scene looks like, or indeed whether they remember any pictorial detail of his appearance. This lack of assessment of memory for visual detail raises a serious question about what this task is actually measuring. Results from studies with patients evaluated for epilepsy surgery indicated that the FP task relies heavily on auditory verbal based cognitive abilities, as well as visual memory, and may better represent a general measure of memory performance (Dulay et al., 2002; Chapin et al., 2009). Lum et al. (2013) showed that differences in the FP task between children with and without specific language impairment was best predicted by a measure of verbal working memory, thus questioning its role as a visual (nonverbal) memory test.

Even the most commonly used visuospatial memory test, the Rey-Osterrieth Complex Figure Test (ROCFT; Osterrieth, 1944—translated by Rey, 1941; Corwin and Bylsma, 1993; Meyers and Meyers, 1996) has been criticized on the grounds of potential for verbal mediation (e.g., Avery, 1990). Although the complexity of the figure may help reduce the value of verbal labelling, some verbal mediation can facilitate design recall (for example, the patient may use verbal cues as to where to draw “the cross,” “the diamond,” or the “Union Jack”).

### The problem of over-abstraction of stimuli

Visual memory tests often use abstract designs or nonsense figures in an attempt to minimize verbal mediation (Lezak et al., 2012). These attempts cannot, however, fully eliminate verbal associations. While use of more complex abstract stimuli may avoid, or at least minimize, verbal mediation (e.g., Continuous Visual Memory Test; Trahan and Larrabee, 1988), it reduces ecological validity, defined by Sbordone and Long (1996) as “the functional and predictive relationship between the patient’s performance on a set of neuropsychological tests and the patient’s behavior in a variety of real-world settings (e.g., at home, work, school, and community)” (p.16), of such tests. As described by Diaz-Orueta et al. (2020), one of the factors with the potential to impact ecological validity in neuropsychological testing is the lack of agreement regarding the specific cognitive constructs measured by a test. For example, the ROCFT can be considered a measure of visuoperceptive processes, visual memory, visuospatial memory, or visuoconstructional abilities, while, at the same time, it correlates with several verbal memory measures. This lack of consensus on what tests actually measure is inextricably linked to the fact that most tests are multifaceted, making it difficult to align any particular cognitive test score to an appropriate cognitive skill (Chaytor and Schmitter-Edgecombe, 2003; Marcotte et al., 2010) and the use of overly abstract stimuli that do not reflect real-world visual memory skills poses a problem in terms of actually measuring how individuals might perform in their daily life. From a clinical perspective, overreliance on abstract stimuli impacts the capacity of neuropsychological test scores to predict real-world performance (Parsons, 2015).

More sophisticated methods of assessing visual recognition memory using same-name alternatives to avoid verbal mediation and over reliance on abstract stimuli have been employed in clinical (e.g., Baddeley et al., 2006) and in research contexts (e.g., Burke, 1987; Burke and Nolan, 1988; Burke and Milner, 1991; Pigott and Milner, 1993; Maguire et al., 1996; Owen et al., 1996) but, with the exception of the Doors subtest from the Doors and People Test (Baddeley et al., 2006), these tools are not readily available to clinicians. However, it should be remembered that even if memory tests succeed in making verbal labelling redundant, memory scores might still be confounded by non-mnestic abilities, such as visual perceptual processing and visuo-construction skills. By way of example, Gfeller et al. (1995) found that a group of patients with constructional impairments scored significantly lower on visual reproduction (Wechsler, 1997b) than did a group with intact constructional skills, despite the groups being matched on a range of other test scores.

### The requirement of a drawing response

Assessment of visual memory often requires a visuomotor response, typically drawing. This is a usual test procedure for widely used tests such as the Benton Visual Retention Test (Benton, 1974), ROCFT (Rey, 1941; Osterrieth, 1944), and the visual reproduction subtests of the Wechsler Memory Scales. According to Barr et al. (2004), many commonly used measures of figural reproduction have been found to be relatively insensitive to the effects of right temporal-lobe dysfunction (see below), and a test like the Brief Visuospatial Memory Test-Revised (BVMT-R; Benedict et al., 1996) seems to lack the necessary sensitivity required for assessing nonverbal memory in this population.

As Lezak et al. (2012) point out, this approach to visual memory assessment can complicate the interpretation of performance as defective performance cannot be attributed exclusively to the memory problems that these tests purport to measure. Indeed, performance may be influenced by failures in processes related to either constructional skills, visual or spatial memory, or to an interaction between these or other factors. Lezak et al. (2012) suggests complementing the assessment with other tests to enable the clinician to estimate the relative contribution of each cognitive process to the final product, referred to satellite conditions in the process-oriented approach methodology (Libon et al., 2013). However, such an approach, while laudable, does not address the issue of construct validity of purported visual memory tests and it promotes lengthy assessment sessions to identify the nature of the patient’s difficulties, instead of promoting the development of a detailed, process-based examination of the construct we are aiming to assess. In addition, for those populations where specific deficits in visuoperceptual and/or visuoconstructional skills may mask the actual performance of memory processes, such as in individuals with Huntington’s disease (Snowden, 2017), Parkinson’s disease (Tröster and Garrett, 2018), Dementia with Lewy Bodies (Metzler-Baddeley, 2007; Tröster, 2008), or Posterior Cortical Atrophy (Crutch et al., 2017), it is even more necessary, for early accurate diagnosis and behavior prediction purposes, to dissociate these cognitive processes and accurately attribute the defective performance to the distinct predominant underlying cognitive dysfunction.

### The problem of sensitivity to specific unilateral brain lesions

Given the criticisms of visual memory tests, it is not entirely surprising that visual memory tests available to clinicians have produced less consistent results in terms of laterality effects compared to verbal memory tests (Brown et al., 2007). There is, however, another potentially more damaging criticism that questions the very construct of visuospatial memory and, consequently, the use of tests designed to measure it as a unitary construct, particularly as a method of detecting unilateral right-sided lesions.

Malec et al. (1991) found that their Visual Spatial Learning Test (VSLT) failed to distinguish between patients with left and right temporal-lobe epilepsy, both pre-operatively and 3 months post-operatively, but Pirogovsky et al. (2015) found the test useful in Huntington’s Disease (HD). More specifically, they found that while recognition memory for abstract designs and memory for object locations remained intact, object-location associations were poor in pre-manifest HD. This test then looks promising in its ability to identify circumscribed memory problems in clinical samples, but the issues related to the use of abstract stimuli, detailed above, remain.

It is clear that at a functional and anatomical level, visuospatial memory can be dissociated into both visual and spatial components. There is clear evidence suggesting that visual and spatial memory represent separate, albeit related constructs. For example, Maguire (1994) and Maguire et al. (1996) found impairments in spatial/topographical memory in patients with both left-and right-unilateral temporal-lobe excisions. However, on a task examining forced-choice visual-recognition-memory for complex scenes, only those with right-sided removals were impaired. Owen et al. (1996), in a positron-emission-tomography study of healthy adults that was designed to compare memory for object features and object locations concluded that: “…in human subjects, memory for object features is mediated by a distributed system that includes ventral prestriate cortex and both anterior and posterior regions of the inferior temporal gyrus. In contrast, memory for the locations of objects appears to be mediated by an anatomically distinct system that includes more dorsal regions of prestriate cortex and posterior regions of the parietal lobe” (Owen et al., 1996, p 9212).

In the same year, Breier et al. (1996) examined delayed memory for the ROCF in preoperative patients with seizures of temporal lobe origin. However, they elected to examine not just the traditional composite score that captures memory for the spatial configuration and figural details together as a single overall score. Rather, they derived two further indices emphasizing separately memory for either spatial or figural aspects of the complex design. As they reported, all three indices distinguished between individuals with right-sided and left-sided seizure onset and, unlike in the left-temporal group, spatial memory was significantly lower than figural memory in individuals with right-sided seizure onset. Furthermore, in individuals with right sided seizure foci, both the spatial and figural memory indices were significantly lower in the presence of hippocampal sclerosis. Taken together, the results suggested that figural memory might be less vulnerable to right hippocampal dysfunction than spatial memory.

Nunn et al. (1999) found dissociations in patients with right-temporal lobectomy, showing that their memory for the location of objects was worse than memory for the objects themselves, despite controlling for the problem of differential sensitivities of the tasks to overall memory impairment. At an individual level, Holdstock et al. (2000) reported on the case of a patient (YR) with relatively selective bilateral hippocampal damage who was impaired on tasks related to spatial memory but exhibited intact visual recognition on standardized memory tests. Additionally, Kessels et al. (2002) showed that there was a double dissociation between patients with left hemisphere strokes (showing impairment on object location binding) and right hemisphere strokes (impaired on positional memory and maze learning), and a specific impairment for spatial-memory tasks in those patients with lesions in the posterior part of the parietal or the occipital lobe; thus showing evidence for selective aspects of memory for object locations.

A similar dissociation of spatial memory and visual object recognition was demonstrated in rodents (Massey et al., 2003). Parallel research suggests that the functional separation of visual and spatial memory may be mirrored anatomically. Research carried out on rodents (e.g., Massey et al., 2003) and on humans (e.g., Smith and Milner, 1981, 1989; Maguire, 1994; Maguire et al., 1996; Milner et al., 1997; Astur et al., 2002) implicated the hippocampus in spatial memory while the temporal cortex and, in particular, the perirhinal cortex, has been found to play a key role in visual object recognition in rodents (e.g., Mumby et al., 2002), monkeys (e.g., Meunier et al., 1993; Nakamura and Kubota, 1996), and humans (Burke and Nolan, 1988; Burke and Milner, 1991; Nakamura and Kubota, 1996; Owen et al., 1996; Simons et al., 2002). More importantly, a growing literature suggests that lateralization effects are dependent on the nature of the material to be remembered. Encoding the visual characteristics of objects relies on right (non-dominant) hippocampal structures and the associated cortical regions while spatial information memory, in contrast, is more dependent on bilateral hippocampal function (Courtney et al., 1996; Maguire et al., 1996; Gotts et al., 2013; Zammit et al., 2017). Of interest, these important distinctions between components of visuospatial materials (and consequently important distinctions between visual and spatial memory) have not always been considered in clinical tests of visual and visuo-spatial episodic memory.

This oversight is apparent when one reviews the history of clinical evaluation of memory detailed below.

### Evolution of visual memory assessment in clinical practice

When one examines the history of the Wechsler Memory Scales, in their various iterations, the difficulties for clinicians in assessing nonverbal/visuospatial memory are clear and, indeed, ongoing (see Kent, 2013, 2016). This examination (see Table 1) of perhaps the most widely used memory assessment test(s) also highlights the problems with visual memory assessments detailed above.

**Table 1:** Wechsler memory scales: history of visual memory assessment and assessment rationale.

	Wechsler Memory Scale (WMS): Version			
	Original (WMS: Wechsler, 1945)	Revised (WMS-R: Wechsler, 1987)	Third Edition (WMS-III: Wechsler, 1997a,b)	Fourth Edition (WMS-IV: Wechsler, 2009a,b) Adult Battery
**Verbal Memory Subtests** ^**1**^	*Logical Memory* (immediate) *Associate Learning* 10-word pairs (six easy, semantically related, four hard, semantically unrelated).	*Logical Memory* (immediate and delayed) *Verbal Paired Associates* (immediate and delayed) Revised name for Associate Learning from WMS. Word-pairs reduced to 8 (four easy, four hard). The procedure matched that of the newly introduced Visual Paired Associates (minimum of three presentations; maximum of six). Delayed trial introduced.	*Logical Memory* (immediate and delayed) *Verbal Paired Associates* (immediate and delayed) Word pairs changed—removing all “easy” pairings. Eight pairs across four trials. *Word Lists* (immediate and delayed) Optional 12-item list-learning test presented over four trials, followed by a new list (interference trial), and short- and long-delayed testing.	*Logical Memory* (immediate and delayed) *Verbal Paired Associates* (immediate and delayed) Number of word-pairings increased to 10 with more “easy” items added to reduce floor effects.
**Visual Memory Subtest(s)**	*Visual Reproduction* The examinee is required to observe, and then draw from memory, a number of abstract geometric designs.	*Visual Reproduction* (immediate and delayed) As per WMS, but with modified content, revised scoring criteria, and the inclusion of a delayed-memory component. *Figural Memory* Requires the test taker to study modular designs for 5–15 s each, depending on complexity, and then to identify the figure(s) from an array in a recognition-memory format. *Visual Paired Associates* Six nonsense designs are each paired with one of six colors for at least three but no more than six learning trials. To achieve “criterion,” the examinee is required to identify all drawing-color pairs (i.e., select the correct color in response to presentation of a specific design), but, regardless of performance, the task is discontinued after six learning trials. The score is calculated from the first three trials and a delayed condition is included.	*Visual Reproduction* (immediate and delayed) Similar in format to WMS-R, but with modifications to the visual stimuli. *Faces* (Immediate and Delayed) Forced-choice recognition—Faces I (immediate) and Faces II (delayed) components. In Faces I, 24 target faces as shown, 1 at a time for 2 s. Then 48 faces (24 targets and 24 distractors) are presented sequentially, and test takers are asked to identify the target faces by responding “yes” or “no” to each face. They are then prompted to keep the target faces in mind for later recognition. Following a 30-min delay, 48 faces (the 24 targets and 24 new distractors) are shown and the task is again to identify the target faces. *Family Pictures* (immediate and delayed) Assesses recall and recognition of complex visually presented information.	*Visual Reproduction* (immediate and delayed)—but re-introduced as core rather than optional. Same items from WMS—III Visual Reproduction; Scoring rules simplified; Recognition testing procedure revised—now uses old visual discrimination format of several items—examinee needs to select correct design; and Optional copy condition introduced to control for visual/ spatial skills. *Designs* (immediate and delayed). Assesses recognition memory for visual details of abstract designs and their spatial locations within an array. Both immediate and delayed testing employed.
**Other Subtests**	*Mental Control* *Digit Span (Forward and Backward)* *Orientation* *Personal and Current Information*	*Mental Control* *Digit Span (Forward and Backward)* *Visual Memory Span* a new subtest designed as a spatial analog to digit span	*Spatial Addition:* Designed as a test of visual working memory. *Letter-number sequencing:* A new subtest designed to assess auditory working memory. A series of interspersed numbers and letters are read aloud to the examinee, and they are required to repeat them in numerical and then alphabetical order.	*Spatial Addition:* *Symbol Span* (Essentially a visual version of digit span) *General Cognitive Screener*
**Key Scores**	*Memory Quotient (MQ):* A single MQ incorporating memory for verbal material (Logical Memory/Associate Learning) and Visual Reproduction is derived.	The WMS-R test scores generate four “memory indices”: as well as an Attention/Concentration Index *General Memory Index:* Logical Memory I, Verbal Paired Associates I, Figural Memory, Visual Reproduction I, Visual Paired Associates I. *Verbal Memory Index:* Logical Memory I, Verbal Paired Associates I. *Visual Memory Index:* Figural Memory, Visual Reproduction I, Visual Paired Associates I. *Delayed Memory Index:* Logical Memory II, Verbal Paired Associates II, Visual Reproduction II, Visual Paired Associates II. *Attention/Concentration Index:* Mental Control, Digit Span, Visual Memory Span.	The core WMS-III test scores generate seven primary “memory indices” as well as a Working Memory Index: *Auditory Immediate Index* Logical Memory I, Verbal Paired Associates I *Visual Immediate Index* Faces I, Family Pictures I *Immediate Memory Index* Logical Memory I, Verbal Paired Associates I, Faces I, Family Pictures I *Auditory Delayed Index* Logical Memory II, Verbal Paired Associates II *Visual Delayed Index* Faces II, Family Pictures II *Auditory Recognition Delayed* Logical Memory II Recognition, Verbal Paired Associates II Recognition *General Memory Index* Logical Memory II, Verbal Paired Associates II, Faces II and Family Pictures II *Working Memory Index* Letter-Number Sequencing, Spatial Span	The core WMS-IV test scores generate four primary “memory indices”: *Immediate Memory Index* Logical Memory I, Verbal Paired Associates I or CVLT-II (Delis et al., 2000) Trials 1–5, Designs I, Visual Reproduction I *Delayed Memory Index* Logical Memory II, Verbal Paired Associates II or CVLT II Delayed Free, Designs II and Visual Reproduction II *Auditory Memory Index* Logical Memory I and II; Verbal Paired Associates I and II Or CVLTII LRN and Delayed Free *Visual Memory Index* Designs I and II, Visual Reproduction I and II *Visual Working Memory Index* Spatial Addition and Symbol Span
**Early critiques (sample)**	Dujovne and Bernard, 1971; Kear-Colwell, 1973, 1977; Russell, 1975; Prigatano, 1977; Lezak, 1983	Brown et al., 1987; Powel, 1988; Loring, 1989; Loring et al., 1989; Chelune et al., 1990; Elwood, 1991; Lezak et al., 2004	Horton and Larrabee (1999); Millis et al., 1999; Tulsky et al., 2003; Lezak et al., 2004; Chapin et al., 2009	Drozdick et al., 2011; Hoelzle et al., 2011; Kent, 2016
**Revisions from earlier edition**	-----	Five major changes made (Wechsler, 1987, p 2): Provision of norms stratified at nine age levels. Replacement of a single global summary score (the Memory Quotient) with five composite scores Addition of new subtests measuring figural and spatial memory. Addition of measures of delayed recall. Revision of the scoring procedures for several subtests to improve scoring accuracy.	Figural Memory, Visual Paired Associates, and Visual Reproduction were replaced with two new tests of visual memory: Faces and Family Pictures Focus changed from material-specific to modality-specific memory	Family Pictures was dropped, in order to meet the design goal of reducing verbalization of visual memory tasks. Faces was dropped because of floor and administration limitations. VR was re-introduced. New visuospatial test (Designs) introduced. Reconfiguration of Index
**Major Critique of visual memory component(s) and component scores**	The *Visual Reproduction* subtest cannot be considered a test of nonverbal memory: simplicity of designs mean they lend themselves readily to verbal mediation Requires a drawing component No delayed memory assessment	*Visual Reproduction* and *VPA* cannot be considered tests of nonverbal memory: simplicity of VR designs and the nature of the VPA stimuli mean they lend themselves readily to verbal mediation. *VR* requires a drawing component *Visual Memory Index* is based on immediate memory alone—and it incorporates the Figural Memory subtest that, according to Loring (1989), appears to assess higher-order visual attention span rather than retention of information over time (i.e., memory). *General Memory Index* is based on immediate memory scores alone, and it fails to distinguish between verbal and visual memory functions. *Delayed Memory Index* fails to distinguish between verbal and visual memory tests—treating delayed memory as a unitary construct. Index scores are composites, and, therefore, subject to criticism.	*Visual Reproduction*—now an optional subtest that does not form part of the core indices. *Faces—*employed a yes/no recognition memory format that was subject to floor effects. It also failed to distinguish between poor memory for faces and “guessing” and poor effort (Chapin et al., 2009). *Family Pictures—*widely criticized because of the extent of verbal mediation. Computation of Index scores continued (see, for example, Visual Immediate, Visual Delayed, Immediate Memory, General Memory). *General Memory Index*: remains a composite of both verbal and visual memory albeit now assessing delayed memory rather than immediate memory as in the WMS-R.	*Visual Reproduction*—cannot be considered a test of nonverbal memory: simplicity of designs mean they lend themselves readily to verbal mediation VR requires a drawing component —that although taken into account to some extent in scoring might still impact performance. *Designs*—Unlike *Visual Reproduction*, DE evaluates spatial memory explicitly, but uses a grid that can facilitate use of a verbal mnemonic to recall locations. Index Scores are computed from a number of test scores.
**Rationale for revision**	From WMS to WMS-R. To introduce new subtests to better balance the assessment of verbal and visual memory. To incorporate delayed memory assessment. To clarify the directions for administration” (Wechsler, 1987, p. 43).	From WMS-R to WMS-III. To address a need to include visual material that is difficult to encode verbally, as well as increase the ecological validity of the instrument (Wechsler, 1997a,b). To reflect more accurately what is being assessed by the subtests. In describing changes in the Indexes, the test developers say: *“There are two notable changes in the index nomenclature. First, the “verbal” label used in the WMS-R was changed to reflect the modality of presentation rather than the index content more accurately. Therefore, the term “auditory,” which is the parallel to “visual,” is used instead of “verbal.”* They then deal with the change in nomenclature, and content, of the Attention/Concentration index of the WMS-R, becoming the Working Memory Index in the WMS-III (WMS-III Manual).	From WMS-III to WMS-IV. One of the stated design goals of the revision was to reduce confounding factors, and, of considerable interest is the fact that amongst the objectives were: Reduce or eliminate motor requirements in administration or scoring where possible; Reduce verbal processing on visual memory subtests; Develop Contrast Scores to partial out confounding cognitive effects (e.g., Spatial Versus Detail; Immediate Versus Delayed); Reduce language level of verbal tasks where possible.	From WMS-IV to its successor: Changes to the WMS-IV (i.e., development of a fifth edition of the WMS) have not yet been outlined by the test developers, but a further update is almost inevitable (see Kent, 2016 for suggested revisions).

1 Re-named as auditory memory subtests in the WMS-III.

In its original format (Wechsler, 1945); visual memory was assessed by means of a Visual Reproduction (WMS-VR) subtest alone. The test taker was required to observe and then, immediately after presentation, to draw from memory a number of simple abstract designs. While one can criticize the WMS-VR subtest on the basis that their simplicity meant the designs lent themselves readily to verbal mediation, that their reproduction required a drawing response, and that there was no assessment of memory following a delay, incorporation of the WMS-VR score into a single Memory Quotient arguably presented the greatest challenge for clinicians who wished to determine the integrity of nonverbal/visuospatial episodic memory. The use of composite or summary scores to characterize performance across a number of distinct tasks or task components has long been criticized as being insensitive to the multitude of specific presentations of brain dysfunction (Prigatano, 1977; Lezak, 1983; Kaplan, 1988). Use of composite scores can mask poor (or good) performance on specific components and it hinders clinical test interpretation.

Despite its limitations, the WMS-VR subtest was retained in the first update of the WMS (WMS-R; Wechsler, 1987), but with the addition of a delayed memory component (WMS-VR-II). Two new subtests (Figural Memory and Visual Paired Associates) were introduced, with the stated intention of securing a better balance of assessment of verbal and visual memory. Arguably, these new subtests did little to enhance assessment of nonverbal/visuospatial episodic memory in any real sense. Figural Memory was an immediate recognition-memory test of abstract designs (there was no requirement to retain the information over time) while Visual Paired Associates paired abstract line drawings with colors in a list-learning paradigm. The examinee was required to learn six design-color associations across repeated presentations, demonstrating their learning after each presentation of the six pairings by pointing to the associated color when presented with an abstract design. Delayed memory for the design-color pairings was also tested—but examinees were not required to reproduce or even identify the relevant design in response to presentation of the associated color. Neither were they required to identify the abstract designs from amongst distractors—thus, memory for the precise visual details of the designs was not actually assessed.

Loring (1989) offered a very critical review of the WMS-R, lamenting the fact that the advancements in cognitive and experimental/clinical psychology over the decades since the publication of the WMS “were largely ignored” (p. 67). In his critique he says: “There exists unquestioned improvement in the test’s “surface structure.” However, the test’s “deep structure,” the area of more theoretical importance and interest, remains essentially unchanged” (Loring, 1989, p. 67) and he argued that “The WMS revision would have been better served by selecting tests that had been previously demonstrated to be selectively sensitive to memory deficits associated with right cerebral dysfunction (e.g., facial recognition; Milner, 1968b; Warrington, 1984)” (Loring, 1989, p. 64). Indeed, Butters et al. (1988) noted, in reference to the WMS-R: “Logical Memory and Visual Reproduction tests were far superior to those from the two paired-associates tasks for differentiating patients from normal subjects” (pp. 145–146). Thus, the inclusion of Visual Paired Associates and the generation of Verbal and Visual Memory Indexes added little by way of content improvement and had real potential to mislead those not well versed in the nuances of index composition. Perhaps Loring’s most damning criticism, however, centers on his assertion that “By including additional visual memory measures, a greater diversity of visual memory functions is sampled. However, the net effect may have been to make this a less material-specific memory measure” (Loring, 1989, pp. 63). As he notes, the new subtests do not appear to be pure measures of visual learning/memory and while likely sensitive to generalized, non-lateralized, memory dysfunction, it is “premature to use the Verbal and Visual Memory Indexes to infer lateralized temporal-lobe dysfunction” (Loring, 1989, p. 67).

Perhaps not surprisingly, many epilepsy surgery centers failed to find that these index scores reliably discriminated lesion laterality (see Lezak et al., 2004). Both focused on immediate rather than delayed memory (which is known to be more sensitive to impacts of TLE) and, as already noted, the extent to which the Visual Memory Index represented nonverbal memory is highly questionable. Similarly, the Delayed Memory Index, wherein delayed memory for verbal material is combined with delayed memory for supposedly nonverbal subtests into a single Delayed Memory Index has presented a mixed picture in terms of clinical findings.

Of interest, both newly introduced visual memory tests were dropped in the subsequent WMS-III edition. Instead, the WMS-III introduced two new visual memory tests (Faces and Family Pictures), with WMS-VR, while retained, consigned to an optional test. Neither new test required a drawing response, a perceived limitation of WMS-VR subtests.

On the surface, it appeared that the WMS-III was a significant improvement on the WMS-R in terms of visual memory assessment, but both subtests proved problematic. Faces, because of the yes/no recognition memory format, failed to distinguish between poor memory and poor “effort” while Family Pictures has been widely criticized because of its heavily reliance on verbal mediation.

As Lezak and colleagues point out “It is surprising that such a verbalizable test as Family Pictures was included as part of the Visual Memory Index, particularly at the expense of Visual Reproduction” (Lezak et al., 2012, pg. 1985). Its inclusion in the visual memory indices is perhaps more easily understood when one considers the change in test focus. In developing this iteration of the WMS, the test developers elected to focus not on a verbal-nonverbal/visuospatial distinction between test groupings (assessing material-specific memory), but rather on modality-specific distinctions (auditory vs. visual memory) without reference to having the visual memory subtests reflect nonverbal visuospatial memory.

From this, it is apparent that the new visual memory subtests introduced into the WMS-III were not designed to represent tests of nonverbal memory. Rather, they were intended to be tests that parallel, in the visual modality, the auditory verbal memory tests. Vanderploeg et al. (2001) provide support for this point when, in discussing limitations of the structural equation modelling (SEM) detailed in the WMS-III Technical Manual (WMS-III: Wechsler, 1997a,b), they note: “Material-specific models were not examined because the WMS-III subtests do not easily lend themselves to that verbal-nonverbal categorization. The visual memory tasks on the WMS-III have a large language component. For example, the Family Pictures subtest requires verbal responses and verbal conceptualization of the visually presented material. In a similar manner, aurally presented tasks are largely verbal in nature, but some individuals likely create visual images to help them learn and retain the material.” (Vanderploeg et al., 2001, pg. 174–175). Given this clear shift in focus, lack of evidence to support a strong association between the Visual Memory Index and lateralized right-hemisphere lesions is not entirely unexpected (Axelrod, 2001).

Somewhat surprisingly, in the most recent edition of the WMS (WMS-IV; Wechsler, 2009a), the test developers elected to drop rather than seek to improve upon the WMS-III Faces and Family Picture subtests. The Visual Reproduction subtest was re-introduced as a core test of visual memory, and a new visual memory test (Designs) was introduced. This latter test set out to assess both visual and spatial memory (Content and Spatial), reflecting a growing number of tests designed for research purposes to capture memory for visual content (or visual detail) and memory for spatial location (spatial information) separately and in combination (object-location binding). This trend reflected the evidence that these two components of visuospatial processing are distinct and utilize different neural networks. Arguably, this subtest represents the first real attempt to assess nonverbal/visuospatial memory in the suite of Wechsler Memory Scales. Despite these improvements, however, a range of criticisms can be made. For example, in terms of clinical data, while a number of clinical samples were included in test development, little attempt was made to evaluate those with unilateral lesions (left-or right temporal lobe epilepsy) and, for most clinicians, the Content and Spatial scores are simply combined into a single DE score without reference to the subtle but important distinctions between the scores.

Table 2 presents an overview of other readily available visual memory tests. The emphasis is on identifying tests of visual or visuospatial memory that are used widely in clinical practice, with a view to considering the extent to which they represent good exemplars of material-specific nonverbal memory tests that might reasonably be expected to be sensitive to the effects of unilateral right hemisphere (temporal-lobe) lesions. Comprehensive detail of the test characteristics, test materials, administration protocols, test scoring, and the impact of demographics on performance measures are contained in a number of seminal textbooks (such as Lezak et al., 2004, 2012; Mitrushina et al., 2005; Strauss et al., 2006; Sherman et al., 2022) as well as in the relevant test manuals. These details will not, therefore, be rehearsed here. Rather, the summary is designed simply to highlight the major features of the tests as they relate to critiques of visual memory test adequacy. Most, if not all, of these tests will be deemed “guilty” of one or more of the following charges, and some will even be found “guilty of all charges”: (1) being susceptible to verbal mediation; (2) relying on overly abstract stimuli; (3) requiring a drawing response; and (4) lacking sensitivity to specific brain lesions.

**Table 2:** Clinical Assessment of visuo-spatial memory.

**Test**	**Brief description**	**Susceptible to verbal mediation**	**Overly abstract stimuli**	**Requires drawing response**	**Sensitive to unilateral and/or specific lesions**	**Notable features**
Benton Visual Retention Test Fifth Edition (BVRT-5) (Sivan, 1992) Original Edition: Benton Visual Retention Test (Benton, 1945)	*Copy, immediate and delayed free-recall of geometric designs.* The BVRT, now in its fifth edition, consists of a stimulus book with three sets of 10 cards containing one or more geometric designs. Each set is an alternative, roughly equivalent, form and each can be administered in a number of formats. Depending on the administration format, the client will be asked to copy the design(s) as accurately as possible (Administration C); to reproduce the design(s) from memory immediately after presentation (either 10 s exposure—Administration A—or 5 s exposure—Administration B) or to reproduce the design(s) from memory following a 15 s delay after an initial 10 s exposure (Administration D).	Yes—many of the designs can be conceptualized verbally	No—many of the designs can be conceptualized verbally	Yes	Mixed findings. Sensitive to brain injury but lacks specificity to unilateral right-hemisphere lesions (Lezak et al., 2012)	Despite its apparent simplicity, this test involves and necessitates many different processes for successful completion. By way of example, Lezak et al. (2012) identified visuomotor response, visuospatial perception, visual and verbal conceptualization and immediate memory span as amongst the component processes tapped by the test. Loads onto visual-perceptual-motor factor primarily, not a pure visual memory factor.
Brief Visuospatial Memory Test—Revised (BVMT-R) (Benedict, 1997) (Barr et al., 2004)	*Immediate and delayed recall of geometric designs and their spatial positions in an array——followed by a delayed recognition-memory for designs test.* Across each of three learning trials, the client views six geometric figures printed in a 2 × 3 array for 10 s and is then asked to draw as many of the figures as possible, in their correct position on a page in the response booklet. A Delayed Recall Trial is administered 25 min later followed by a Recognition Trial, in which the respondent is asked to identify the six target stimuli and six distractors in a yes/no format. An optional Copy Trial may be administered to screen for severe visuoconstructive deficits and to help in scoring and, presumably, interpreting responses on the recall trials.	Yes—many of the designs can be conceptualized verbally	No—many of the designs can be conceptualized verbally	Yes	Mixed findings. Sensitive to brain injury but lacks specificity to unilateral right-hemisphere lesions. By way of example, Barr et al. (2004) reported that learning, delayed recall, or yes/no recognition scores failed to differentiate between left and right TLE patients. They concluded that the BVMT-R does not appear to have the sensitivity required for assessing nonverbal memory in this population.	Memory for visual detail and memory for spatial location are not examined separately. Scores for each item freely recalled range from 0 to 2 depending on whether the drawing is accurate and in the correct location. A composite score is, therefore, obtained. Six equivalent, alternate stimulus forms.
Complex Figure Test (Rey, 1941; Osterrieth, 1944; Meyers and Meyers, 1996)	*Copy and free recall of a complex geometric Figure.* A complex geometric figure is first copied, and then, depending on the administration format, is followed by immediate and delayed free-recall trials.	Yes	Yes—There is a clear potential for some verbal mediation—but the complexity of the stimulus is such that it cannot be remembered by means of a verbal strategy alone.	Yes	Mixed findings. Breier et al. (1996): right temporal epilepsy patients with hippocampal sclerosis were impaired compared to left, those with no hippocampal sclerosis were not.	A number of administration and scoring formats are used, making direct comparison across test centers and across research studies difficult.
Continuous Visual Memory Test (CVMT) (Trahan and Larrabee, 1988)	*Recognition memory for “new” and “repeated” abstract designs.* Consists of 112 abstract designs exposed sequentially for 2 s with seven target figures repeated six times. The task is to discriminate the new stimuli from the repeated stimuli. The Total score is the number of correct “new” and “old” responses.	Some—but unlikely given the number of stimuli.	Yes	No	Mixed findings. Both right- and left-lateralized stroke patient groups performed more poorly than controls (Trahan et al., 1990) but impaired performance was more prevalent following right-sided lesions. The CVMT did not discriminate between right and left temporal-lobe epilepsy seizure onset (Snitz et al., 1996).	Overall cognitive function and visuoperceptual processing were related to CVMT scores (Snitz et al., 1996).
Doors and People Battery—Doors subtest. (Baddeley et al., 1994)	*Recognition memory for visual features of meaningful stimuli (Doors).* In both parts of the test (Part A and the more difficult Part B), 12 color photos of doors are each shown individually for 3 s, followed by testing recognition memory for each door in a four-alternative forced-choice paradigm. Each target door is presented together with three distractor doors that vary, across the two parts of the test, in terms of similarity and, as a result, difficulty.	No: Although the stimuli can be named (i.e., door), the benefit of the verbal label is rendered meaningless in the context of forced choice recognition memory for same-name-alternatives.	No	No	Verbal memory functioning was significantly more impaired in patients with left temporal lobectomy (lTL), whereas visual memory was more impaired in right TL (rTL) patients (Morris et al., 1995)	Although immediate visual recognition memory is assessed, spatial memory is not.
Doors and People Battery—Shapes Test (Baddeley et al., 1994)	*Immediate and delayed free recall of geometric designs.* Four simple line drawings are presented sequentially for 5 s each, with immediate and delayed recall trials. The subject copies each stimulus, and subsequently attempts to draw them from memory. A total of three learning trials are allowed, followed by a delayed recall.	Yes—many of the designs can be conceptualized verbally	No—many of the designs can be conceptualized verbally	Yes	Mixed findings	
Rivermead Behavioural Memory Test—Third Edition (RBMT-3) (Wilson et al., 2008) The original RBMT was published in 1985, with an update (RBMT-II) in 2003.	*Visual memory is assessed in a number of distinct subtests.* Picture Recognition—Delayed Recognition Face Recognition—Delayed Recognition Route—Immediate Recall Route—Delayed Recall	Yes	No	Depends on subtest.	Mixed findings. Several studies have shown that the RBMT is a valid instrument for detecting everyday memory problems in clinical samples.	RBMT-II included an update of materials only, e.g., included more multiracial stimuli to reflect ethnic diversity of UK. RBMT-3 includes new items on tests, new materials, a new subtest (Novel task) and increased normative sample
Ruff-Light Trail Learning Test (RULIT) (Ruff and Allen, 1999)	*Nonverbal route-learning task* This test was designed to minimize (or eliminate) verbal mediation. It requires the individual to learn a specific 15-step pathway through circles on a sheet of paper. The circles are interconnected by lines and at each circle along the pathway, there are three to five choices for the next step, only one of which is correct. At each choice point, the tester indicates whether a choice is correct, and the individual continues making selections until the correct choice is made. This process continues through the 15-step trail. Repeat trials are administered until the path is recalled without error on two consecutive occasions (up to 10 trials).	Potentially	No	No	Mixed findings. According to the test publishers, the RULIT is a psychometrically sound measure of visuospatial learning and memory that is sensitive to right-hemisphere functioning (Allen and Ruff, 2007)	Does not require drawing skills, good eyesight, good motor control, neither does it require a high degree of visuospatial integration.
	Immediate memory is assessed by means of the number of steps correctly completed in Trial 2 (i.e., after the first presentation of the complete trail). Learning is assessed by means of the number of trials required to master the task and the number of errors across Trials 2–10 (or until the task is mastered) while long-term retention is assessed by means of a 60-min delayed recall.				Araujo et al. (2009) found that presurgical patients with right TLE had significantly *better* scores than patients with left TLE suggesting that the RULIT may not be an appropriate test for presurgical epilepsy evaluations.	Tests spatial memory but does not assess memory for visual details.
Shum Visual Learning Test (SVLT) (Shum et al., 1999)	*Non-verbal learning test for abstract designs evaluated by recognition memory.* Format is similar to the RAVLT. On each of the 5 learning trials, 10 target stimuli (Chinese characters) are displayed sequentially for 2 s each. Following each presentation of the 10 stimuli, recognition memory is tested. A second character set, designed to measure interference, is then presented followed by a recognition-memory trial. Finally, a recognition trial for the original set is undertaken.	No—uses Chinese characters as stimuli as these are not easily verbalized by individuals who do not read Chinese.	Yes	No	No studies on TLE specifically have been reported	
Visual Spatial Learning Test (VSLT) (Malec et al., 1991)	*Visuospatial paired-associate learning test with seven pattern-location pairings.* The VSLT consists of a 6 × 4 grid and 7 different nonsense designs that are, according to Lezak et al. (2012) truly difficult to verbalize. After seeing the designs placed on the grid, clients are given an empty 6 × 4 grid and 15 designs. Their task is to select the target 7 designs and to place them in the original grid position. In total, VSLT consists of five learning trials followed by a 30-min delayed recall trial.	Potentially. Although correlated with the WMS Visual Reproduction, the VSLT also had correlations that fell within similar range with some verbal memory tests.	Yes	No	Mixed findings	Factor analysis failed to demonstrate the VSLT as a measure of nonverbal memory distinct from verbal memory (Smith et al., 1992).
	Performance is scored for recognition learning of the designs, recall of the target positions on the grid and recall of designs in their proper places on the grid.					
Warrington Recognition Memory Test (WRMT)—Recognition Memory for Faces (Warrington, 1984)	*Forced-choice recognition memory for faces.* 50 male faces are presented sequentially for 3 s each and the client is required to make a judgement about whether the face is “pleasant/unpleasant”—designed to ensure that each face was processed at least to some degree. Recognition memory is assessed immediately after presentation of the 50 faces, wherein each target face is paired with a similar distractor.	No	No	No	Both left and right lesions impaired, but no significant difference between the two. (Warrington, 1984)	The face stimuli are now dated and do not reflect cultural diversity. There are also questions about the adequacy of the normative data.
Wechsler Memory Scale —Faces Subtest (Wechsler Memory Scale—Third Edition—WMS-III: Wechsler, 1997b)	*Immediate and delayed recognition memory for unfamiliar faces* This test of facial recognition memory is similar to the faces subtest in Warrington’s Recognition Memory Test—but a yes/no rather than forced-choice paradigm is used. 24 unfamiliar faces are shown in sequence at a rate of one every 2 s. Recognition memory is assessed immediately after exposure to all of the faces. The 24 target faces are shown sequentially, interspersed among 24 foils, and the client’s task is to identify which faces had previously been studied. Delayed recognition memory is tested with the 24 target faces and 24 new foils.	No. As Lezak et al. (2012) note, it is difficult to use verbalization to encode a large number of faces presented briefly.	No	No	Questionable. Doss et al., (2004) reported that patients with right temporal lobectomies performed worse on Faces than on WMS-III Logical Memory (LM) and Verbal Paired Associates (VPA) while the reverse was true for a group with left-temporal lobectomies.	This test was plagued by high guess rates and was dropped from the Wechsler Memory Scale—Fourth Edition (WMS-IV). Poor correlation with other visual memory test scores, suggesting it may measure a different aspect of visual memory or that it allows for a high guess rate (Millis et al., 1999).
Wechsler Memory Scale—Visual Reproduction (Wechsler, 1945, 1987, 1997b)	*Immediate and delayed free recall of geometric designs.* Visual Reproduction was originally developed as an immediate free-recall test for geometric designs, with a delayed recall trial added subsequently. The second revision of Visual Reproduction (VR-III) (Wechsler, 1997b) included a simple design to lower the floor of the test, deletion of a design from earlier editions and a a slight modification to one of the stimuli from the original scale. On the WMS-III, four items (three single figures and the fourth with two geometric designs) are presented for 10 s each. Immediately after presentation, clients are required to draw what they remember, with delayed free-recall assessed following a 30 min delay. A 48-item recognition memory test and a seven-item discrimination test were added, as optional extras, to identify differences in recall and recognition capacities and a copy task can also be administered to examine the potential role of motor difficulties.	Yes—the relative simplicity of the designs encourages verbal encoding	No—many of the designs can be conceptualized verbally	Yes	Findings for patients with lateralized TLE have been mixed (see Barr, 1997; Lezak et al., 2012), likely reflecting the fact that the stimuli can be encoded verbally. Proven highly sensitive to cognitive deterioration associated with dementia (Wang et al., 2009)	Despite its long history of use, dating back to the early WMS versions, VR, although retained, is not considered a core subtest of the Wechsler Memory Scale—Third Edition (WMS-III)
Wechsler Memory Scale—III Family Pictures (Wechsler Memory Scale—Third Edition—WMS-III: Wechsler, 1997b)	*Immediate and delayed verbal recall of “complex, meaningful, visually presented information”* Following familiarization where clients are “introduced” to seven members of a family (mother, father, grandmother, grandfather, son, daughter, dog), four pictures are each shown to the client for ten seconds. Memory for each scene is tested using free recall for the four actors from a family, free recall of what they were doing in the scene, and identification of their location in a 2×2 grid. Immediate and delayed recalls are obtained.	Yes—the test is highly verbalizable. (see Lum et al., 2013)	No	No	Mixed findings. FP does not discriminate lesion laterality effectively. Chapin et al. (2009) reported that change in FP score did not differ post-surgically for left- vs. right TLE patients and they concluded that, with both verbal and visual encoding, FP is minimally sensitive to lateralization of temporal lobectomy. Dulay et al. (2002) similarly found no difference in FP performance in groups of patients with left vs. right TLE.	Family Pictures is considered a “visual analogue to the Logical Memory subtest” (Wechsler, 1997b, p. 15). Given its nature and characteristics, it would appear that it was not developed to be a nonverbal visual memory task; rather, it was designed to assess memory for complex meaningful material presented visually. Notably, Family Pictures was not retained in the WMS-IV (Wechsler et al., 2009), either as a core or as an optional subtest.
**Wechsler Memory Scale—Fourth Edition (WMS-IV)—**Visual Reproduction (Wechsler et al., 2009; Wechsler, 2009a, 2009b)	*Immediate and delayed recall of abstract geometric designs.* In this current form, the same designs and administration as WMS-III is retained but the scoring rules are simplified. In addition to immediate free-recall, clients are told that they will be asked to again draw the designs from memory following a delay. Delayed recall may be followed by a recognition memory test of each design. Recognition testing procedure was revised—now uses old visual discrimination format of several items—examinee needs to select correct design; Optional copy condition introduced to control for visual/ spatial skills.	Yes—the relative simplicity of the designs encourages verbal encoding	No—many of the designs can be conceptualized verbally	Yes	Mixed findings. Findings for patients with lateralized TLE have been mixed (see Lezak et al., 2012), likely reflecting the fact that the stimuli can be encoded verbally.	Although undoubtedly an improvement from earlier editions, there are ongoing problems with the WMS-IV—such as the unexplained shift in approach (e.g., from a material-specific to a modality-specific model of memory—and back again), its underlying factor structure, and the adequacy of its visual memory tests.
**Wechsler Memory Scale—Fourth Edition (WMS-IV)—***Designs* (Wechsler et al., 2009; Wechsler, 2009a, 2009b)	*Visuospatial learning task; abstract designs are paired with specific spatial locations.* Assesses recognition memory for visual details of abstract designs and their spatial locations within an array. Both immediate and delayed testing employed.	Potentially—a verbal mnemonic might be used to recall spatial locations.	Yes	No	Mixed findings. See, for example, Bouman et al. (2016)	Relatively small number of spatial locations on the presentation and test grid. Composite scores are derived combining Content and Spatial scores and combining scores from this test with others. Pinjala (2020) questions the extent to which visual content memory is actually assessed.

Even this cursory overview serves to illustrate the limitations of current clinical assessment tools. Arguably, only a small number of tests detailed here (e.g., Doors and People Battery—Doors subtest, Baddeley et al., 1994; Warrington Recognition Memory Test (WRMT)—Recognition Memory for Faces, Warrington, 1984; DE: WMS-IV, 2009) might be considered good exemplars of nonverbal visual memory and even these are subject to criticism. We now turn our focus to novel approaches to visual memory assessment and the potential opportunities these present for clinical practice.

## Overcoming existing challenges: Novel research test developments and clinical opportunities

A number of visual memory tests have been developed by researchers [see for example: Kimura’s Recurring Figures Test (RFT), Kimura, 1963; Continuous Recognition Memory Test (CRMT), Hannay et al., 1979; Computerized Visual Recognition Test, Flicker et al., 1987; Biber Figure Learning Test, Glosser et al., 1989; Spatial Array Memory Test (Meador et al., 1990), 7/24 Spatial Recall Test (Rao et al., 1984; Gontkovsky et al., 2004), (Spatial Location Test, Sanchez et al., 1997); The modified Location Learning Test, Kessels et al., 2006; Indiana Faces in Places Test, Beglinger et al., 2009; VisMET, Haque et al., 2019; Visual Association Memory Test, Huang et al., 2019; Visual Memory Test based on Snodgrass Pictures (VMT-SP), Muñoz-Machicao et al., 2019; The Continuous Visual Memory Test-Update and Extension, Henry, 2021; Evaluation of Novel Cognitive Assessment System for Testing Visual Memory of the Elderly, Lin et al., 2021; Short Digital Spatial Memory Test, Poos et al., 2021] but these tests are not readily available to clinicians, and they have not been generally been validated in clinical samples. Others have developed test instruments to address specific research questions, typically within the context of temporal-lobe epilepsy. Notably, these research-based tests differ in the extent to which they assess memory for visual details or memory for spatial locations.

In an early study examining the effects of unilateral anterior temporal neocorticectomy carried out in Dublin as a treatment for intractable epilepsy (see Hardiman et al., 1988; Burke and Staunton, 1993; Widdess-Walsh, 2007), Burke (1987) sought to overcome the problems of verbal mediation, the use of abstract stimuli and the requirement of a drawing response widely used in visual memory tests. Using a forced-choice visual recognition memory paradigm, she required patients to identify previously seen line drawings of common objects from a same-name-alternative. In this manner, she sought to evaluate memory for the precise visual details of the presented stimuli. The test results suggested that lateral infero-temporal regions supported memory for the visual features of objects (Burke, 1987; Burke and Nolan, 1988). Subsequently, she demonstrated that visual-recognition-memory deficits were not exacerbated by encroachment upon the hippocampus (Burke and Nolan, 1988; Burke and Milner, 1991).

Experimental tasks looking at memory for object locations have used visually presented scenes and arrays. They have tested memory for the spatial aspects of that information either through altering the material and requiring the person to identify the change (e.g., Pigott and Milner, 1993), or through requiring the person to attempt to reconstruct the spatial array of objects that they had previously seen (Smith and Milner, 1981, 1989; Baxendale et al., 1998; Bucks et al., 2000; Kessels et al., 2010). These tests, like many experimental tests of visual memory, are not readily available to clinicians. Most importantly, no single test is currently available clinically that permits the clinician to evaluate both visual recognition memory (other than for abstract stimuli) and memory for object locations within the context of a single test.

In the absence of a test that permits the clinician to evaluate both visual-recognition-memory and memory for object locations (other than for abstract stimuli), the What-Which-Where Test (WWW-T) was developed (Gallagher, 1998, 2003; Burke and Gallagher, 2000; Burke et al., 2018). The WWW-T was a theoretically motivated instrument that represents an attempt to dissociate visual and spatial components of visuo-spatial memory. One of the primary aims in developing the test was to incorporate both visual-recognition-memory and memory for object locations, two key aspects of visuo-spatial memory, within the context of a single test. In developing the test, the authors set out to overcome the limitations of existing purported nonverbal memory tests that used abstract stimuli with or without a memory for location component by assessing memory for spatial location and memory for figurative details of concrete objects using a paradigm that rendered the use of verbal labels redundant. This approach of using same-name-alternatives (SNAs) as foils for previously viewed concrete objects in a recognition-memory paradigm had previously been adopted to assess the impacts of unilateral left-and right-temporal neocorticectomy (Burke, 1987; Burke and Nolan, 1988; Burke and Staunton, 1993; Maguire et al., 1996), in comparing the impacts of unilateral anterior temporal resections confined to lateral temporal cortex and those that encroached on mesial temporal structures (Burke and Milner, 1991) and with healthy controls when examining memory for figurative detail and spatial location (Owen et al., 1996).

The WWW-T entailed the presentation and study of a three-dimensional (3-D) doll’s house living-room, containing items of scaled furniture. Individuals are required to (1) recall what the items were (room-inventory or “What” was present), (2) identify the inventory items from amongst SNA distractors (identify the items based on figurative detail: “Which” items), and (3) to recall the spatial location in which items were presented (“Where”). The “What” subtests involve what are considered to be a dual-coding task (see Paivio, 1986), while the “Which” and “Where” subtests were designed to be, respectively, material-specific measures of visual recognition and of spatial recall. Because of their clinical relevance and reflecting the importance of a process-oriented-approach to neuropsychological assessment (Kaplan, 1988; Poreh, 2006; Ashendorf et al., 2013; Libon et al., 2013; Diaz-Orueta et al., 2020; Blanco-Campal et al., 2021), the WWW-T offers an operational definition of the distinct cognitive processes of visual-recognition-memory and memory for object locations by way of evaluation and scoring of discrete sub-components of the test (Gallagher, 1998, 2003; Burke and Gallagher, 2000; Burke et al., 2018).

**Figure 1:**
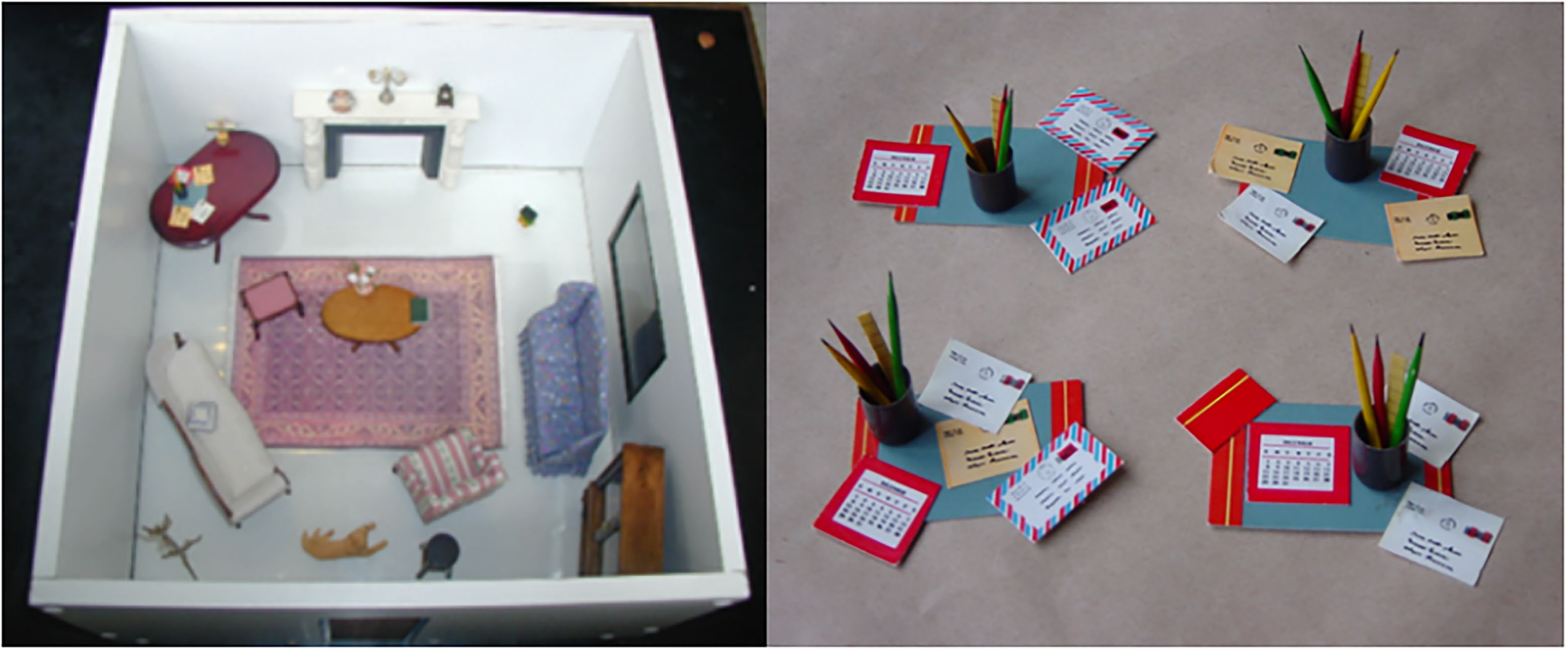
The target items from the WWW-T, as presented to participants, together with the four same-name alternative versions of the “desk-set” used in the visual recognition (figurative detail—Which) subtests of the 3-D WWW-T. Reproduced with permission from Dr. Colin Gallagher and Prof. Teresa Burke.

The 3-D WWW-T consists of a miniature room in which are placed items of doll’s house furniture. Of the 21 items within the room, one represents an anchoring item (a table upon which other items can be placed), while the others serve as target items for the item recall (inventory) and the visual recognition (“What” and “Which”) components of the test. A further 60 items serve as foils (three for each target item) for use in the visual recognition subtests (“Which” component). Each item and its three foils shared the same verbal label (i.e., were SNAs). Thus, a four-alternative forced-choice recognition memory test format (target and three foils) was used to assess memory for figurative or visual detail (see Figure 1).

The 21 items (one anchor, 20 target items) were set up in the miniature room and this room was presented to the participant. Each item had to be named, following a pre-set order (see Gallagher, 1998, 2003; Burke and Gallagher, 2000; Burke et al., 2018, for further detail of item and foil selection). A maximum of 20 s was allowed for each item to be named and after all items were named, the room and its contents were removed from the participant’s view. Thirty seconds later, the person was asked to recall the names of the items contained within the room with a total of 2 min allowed for this subtest corresponding to the “What” component of the WWW-T (Item Recall I). Next, the person was presented, in turn, with four same-name-alternative (SNA) versions of each of the 20 items (Table A—the large corner table, the anchor item, was not included in this subtest) and they had to identify which versions they had seen previously (the “Which” component of the WWW-T—Figurative Detail I). If the participant was unsure, they were encouraged to guess. The testee was then presented the empty room and was required to place each of the 20 target items in its original position (the “Where” component of the WWW-T - Location Recall I). This subtest followed a pre-set order for replacing the items because the test design was such that some items had to be placed on top of others. Delayed recall of the names of the target items followed, before delayed recognition-memory for figurative detail (Figurative Detail II) and delayed recall of object location (Location Recall II). Preliminary factor analytic studies indicated that the WWWT was not merely tapping nonverbal memory, but that it was actually measuring two separate aspects of nonverbal memory. Furthermore, these aspects of nonverbal memory (recognition memory for figurative detail and recall memory for object locations) appear to be distinct from what is measured by the ROCFT. This poses serious issues for the assessment of visuo-spatial memory in clinical practice, as it suggested that the visual and spatial components of nonverbal memory may, in fact, be separate constructs, a suggestion that is supported by the clinical and research literature. The conclusion to be drawn from this is that visual and spatial memory should not be treated as the single concept of visuo-spatial memory.

In terms of potential development as a clinical tool, the WWWT, and other tests that might adopt a similar methodology, avoids a number of the problems identified with other visual memory tests.

1. The use of SNAs in the visual recognition part of the test diminishes the utility of verbal memory (verbal labels) as an aid to memory of the objects. Additionally, the use of a relatively large number of objects and a room with no internal grid reduces the potential for verbal mediation in the object-location component of the test. This contrasts with tests such as the 7/24 Spatial Recall Test (Rao et al., 1984) and the Design subtest of the WMS-IV (Wechsler, 2009b), where a number mnemonic may be used to aid test performance.
2. The test’s use of concrete items of furniture set up in a room in a fashion that mimics real-world relations between objects avoids the problem of over-abstraction.
3. Unlike many other tests of visual memory, the test does not require a drawing or constructional component. In fact, even patients with constructional difficulties or muscular impairments severe enough to prevent them from replacing the items themselves can still be tested. In such cases, the clinician/test administrator can replace the items, with the person directing them where each item should be replaced.

In summary, the methodology employed in the WWW-T avoids a number of the criticisms that have been leveled at some commercially available visual memory tests. Preliminary results also suggest that the test may be tapping into the two distinct aspects of nonverbal memory (memory for figurative detail and memory for spatial location), as opposed to a single visuo-spatial memory construct. This is consistent with a growing literature demonstrating dissociation between visual and spatial memory on delayed memory tests and studies demonstrating a reliable crossed double dissociation of a visual and a spatial component in the short-term retention of single stimuli (Klauer and Zhao, 2004).

At present, the WWWT test has not been examined in sufficient detail to be considered for more widespread use and, in its current formats, it is not likely to appeal to clinicians because of the need for manual recording and scoring of responses. Current research directions with this test include its development as a computerized test to facilitate administration and scoring. Obviously, a more detailed examination of its underlying constructs and an examination of its potential utility in clinical settings would also be required. Surprisingly, despite the ready availability of computer software designed to facilitate the design of spatial memory tests (see Object Relocation Task; Kessels et al., 1999), a test that captures simultaneously memory for spatial location, memory for visual details (other than for abstract designs or for faces) and the binding of these stimuli to locations has not yet been developed. Additionally, inspired by recent studies such as the one by Mazurek et al. (2015) in their development of their what-when-where task in real life settings (see below), future attempts will be made to further increase the ecological validity of the tool by means of transforming the test into a Virtual Reality environment that may then approach visual recognition memory, memory for object location and their binding in a more accurate, reliable and ecologically valid way.

Another test to overcome the over reliance in abstract stimuli comes from Beglinger et al. (2009), who have shown preliminary results for a novel visual memory test, Indiana Faces in Places Test (IFIPT), that is motor free, uses faces as stimuli instead of abstract figures, and contains learning trials and an incidental recall trial to reportedly examine visuospatial memory. The test comprises 10 target black-and-white faces paired with 10 spatial locations represented by boxes on a page. In this preliminary study, the IFIPT showed moderate test–retest reliability and correlated moderately with other visual (non-facial) memory measures. It also showed clinical utility in discriminating between a sample of normal controls and participants with Mild Cognitive Impairment. Although promising as tool for assessment of visuo-spatial memory, this test, like the WWW-T, requires further research and validation before it might be employed in a clinical context.

Similar results have been reported by Pertzov et al. (2012), who supported the existence of distinct memory representation for location and identity of objects, and showed how participants in an object location task could “swap” the correct location and identity of objects held in memory, in a manner that could not be explained by forgetting of object identity or location alone, but rather appeared to arise from failure to bind object identity and location in memory, showing that instead of forgetting objects completely, it is the links between identity and location that are prone to be broken over time.

Mazurek et al. (2015) developed the What-Where-When (WWW) memory test, described by them as a novel, more ecologically valid test of episodic memory (compared to other episodic memory tests) adapted from Holland and Smulders (2011). In the first of two test sessions, participants were required to hide eight common objects in pre-determined locations in a cluttered office. The objects were presented sequentially, and the locations were identified by the examiner in a predetermined sequence. During this time, the subject, who remained naïve to the test purpose, was repeating a sentence aloud. The second test session took place on average 2 h later; participants again performed the same task, placing a second set of objects in new predetermined locations. Finally, sometime later, the participants returned to the room in which they had hidden the objects and were asked to recall which objects were hidden (What) in which locations (Where) and on which of the two occasions (When). According to the authors, the data suggest that the WWW memory task draws on similar processes to other episodic memory tasks. They go on to say, “The design of the task (remembering real objects, incidentally memorized in a real-world environment) additionally increases its ecological validity over existing tasks, making it potentially a better test of their practical memory skills.” (p. 13). Surprisingly, memory for the visual details of the visual objects was not assessed.

The V-SMART (Vik et al., 2018) was developed to address perceived limitations of the VSLT (Malec et al., 1991) detailed above, but the clinical utility of the test has not yet been established. This test, however, like the VSLT, relies on abstract stimuli to assess recognition memory for visual material, likely reducing its “real” clinical utility.

More recently, Robinson et al. (2018), using the Memory Circle test (by means of which participants are presented with a circle comprising 12 sectors, each containing a line drawing of an easily recognized object, for 30 s, and then they are presented with a blank circle showing the sectors and they have to recall the names of the objects into the correct sectors) found that memory for where objects are located proved a better predictor of later Alzheimer’s Disease pathology than memory for what those objects are. This is consistent with reports of topographical memory impairments in MCI and early AD and network-level degeneration incorporating posterior cortical as well as medial temporal structures (Pengas et al., 2012).

## The role of computerized testing and virtual reality: Can technology help overcome the challenges of neuropsychological assessment of nonverbal visual/visuospatial memory?

### Recent history of technological developments for visual/visuospatial memory assessment

Over the course of the last 25 years, several attempts have been made to develop computerized tests that would permit an accurate neuropsychological assessment of visual and spatial aspects of memory. Some have focused on developing computerized versions of already existing tests while others have developed new tests with the stated purpose of assessing visual memory. Beblo et al. (2004), for example, developed the Block Suppression Test, which is essentially a computerized version of the Corsi test. Some of these developments, like the electronic version of the Corsi test (the Modified Corsi Block-Tapping Test, MCBT) and the Modified Walking Corsi Test (MWCT), based on a wireless “Magic Carpet”—a set of tiles that are lit by a computer allowing a large variety of possible combinations of sequences (Perrochon et al., 2014), have been carefully developed and have proven efficient in experimental settings to differentiate between healthy older adults and individuals with MCI. Wang et al. (2013) also developed and tested a computerized test known as Modified Spatial-Context Memory Test (SCMT) to differentiate participants with amnestic mild cognitive impairment (a-MCI) from those with mild dementia of Alzheimer’s type (m-DAT) and normal controls by modifying an existing test of spatial context memory (SCMT) designed so as to evaluate the function of brain regions affected in early m-DAT. Their test showed high sensitivity and specificity in discerning those with a-MCI from normal population, although it was relatively ineffective in discriminating a-MCI patients from those with m-DAT.

More widely known is the *Cambridge Neuropsychological Test Automated Battery* (CANTAB), a computerized suite of tests, developed for research purposes, that facilitates assessment of functioning within a number of domains (memory; attention and psychomotor speed; executive function; and emotion and social cognition; CANTAB: www.cambridgecognition.com; Sahakian and Owen, 1992; Robbins et al., 1994; CANTAB®, 2019). Within the memory domain, CANTAB provides a suite of tests designed to assess different components of both verbal and visual memory. Aspects of verbal memory are assessed by means of: Digit Span (DS), assessing verbal short-term and working memory; Verbal Recognition Memory (VRM), assessing verbal memory and new learning of a list of words *via* free recall and recognition memory; Verbal Paired Associates (VPA), designed to assess learning and memory for eight word-pairs; and Digit Span (DS), designed to measure short-term and working memory for verbal material. Within the visual memory domain, these tests are: Delayed Matching to Sample (DMS), designed to assess visual-matching ability and short-term visual recognition memory (0, 4, or 12 s delay) for patterns that do not lend themselves readily to verbal labels; Paired Associate Learning (PAL), designed to assess visual memory and new learning; Pattern Recognition Memory, designed to assess visual pattern recognition memory in a two-alternative forced-choice paradigm; Spatial Span (SS), designed to assess visuospatial working memory capacity. Of these visual memory tests, PAL and PRM offer clinicians the best opportunity to assess visual memory for nonverbal materials. The clinical utility of PAL has been demonstrated in the assessment of dementia (Fowler et al., 2002; Barnett et al., 2016), while its sensitivity to hippocampal lesions has also been reported in Mild Cognitive Impairment (MCI; Égerházi et al., 2007; de Rover et al., 2011; Meyer et al., 2013; Nathan et al., 2017) and just recently, Pettigrew et al. (2022) have suggested that PAL may be more sensitive to amyloid positivity as measured by Positron Emission Tomography than are a range of other standard neuropsychological tests.

The PRM is a visual pattern-recognition memory test in which a series of abstract visual patterns designed to be difficult to name is presented, sequentially, in the center of a computer screen. Following presentation, recognition memory is assessed. Like PAL, the PRM has been validated clinically but its sensitivity to unilateral right-temporal damage remains unclear.

Beyond these classic computerization paradigms, Haque et al. (2019) presented a highly innovative approach to obtain a “purer” test to evaluate visual and visuospatial aspects of memory based on eye-tracking technology. They developed “a passive, efficient, and sensitive paradigm” (p. 93) that aims to evaluate visuospatial memory in individuals with memory impairment and in healthy controls. They describe the development of a Visuospatial Memory Eye-Tracking Task (VisMET) that is based on eye movements to estimate actual visuospatial memory performance. In their research, participants were requested to observe a series of naturalistic images followed by the same set of images with either an object removed, or a new object added, with the idea of altering the visuospatial relationships between the objects and locations. As a measure of memory, the amount of time that participants spent viewing these manipulations (which could consist of an added new object and location-i.e., added condition-or on a removed object and location that was previously viewed-i.e., removed condition) was measured. The sample of participants comprised 40 individuals with Alzheimer’s Disease Dementia, 74 with MCI and 182 healthy controls (total *n* = 296). Results showed that healthy controls were the ones spending significantly more time observing these “changes” or manipulations compared to the clinical groups, and this variable (i.e., amount of time spent viewing these manipulations) could be used as a predictor of cognitive impairment and disease status, hence, making the ViSMET an efficient paradigm to detect “objective,” visual memory impairment without relying on verbal responses or explicit visual recognition answers. This paradigm avoids the need for an explicit response and awareness of performance deficits, therefore minimizing the possibility of frustration or even distress in impaired subjects who may discontinue the task and decline future assessments.

### The relevance of virtual reality for visual/visuospatial memory assessment

Virtual reality (VR) offers a potentially interesting alternative for the assessment of many cognitive processes. VR reproduces 3-D environments in which the person undergoing assessment interacts in a dynamic way with a sense of immersion in that environment similar to the presence and an exposure to a real environment (Diaz-Orueta et al., 2014). Although development of VR technology was initially modest and costs were high (Tarr and Warren, 2002), VR technologies are now readily accessible for use in research and clinical contexts. As Smith (2019) points out, VR allows researchers to strike a balance between ecological validity and an adequate amount of experimental control and can potentially enhance both the verisimilitude (how well as task simulates a real-life situation) and veridicality (how well the results reflect the issue at hand) of a task. In order to be considered VR, one must be able to interact with the environment in real time, with as little delay as possible between the user’s action and the response of the environment.

One of the main features of VR is immersion. According to Tijs (2006), immersion can be roughly defined as the extent to which a player is “into” the virtual environment, and it can comprise different dimensions such as emotional involvement, curiosity, spatial dissociation (transportation), temporal dissociation, heightened enjoyment, sense of control, and focused attention. As Smith (2019) states, most studies show a pattern of better episodic memory performance in more immersive systems. However, immersion alone is not enough to develop an appropriate environment for neuropsychological assessment.

Visual fidelity (how well a VR system reproduces the visual aspects of a real-world environment) is another asset, and it comprises details like color, texture, and lighting effects. For example, Rauchs et al. (2008) found that reduced visual fidelity during retrieval led to less efficient and accurate spatial navigation; Ragan et al. (2010) found that higher fidelity for field of vision and field of regard both increased memory performance. Audio fidelity is also important to consider when developing a VR environment. Davis et al. (1999) created a VR study with high-fidelity, low-fidelity and no sound and found that the higher the fidelity the better participants’ memory performance was. Lin et al. (2002) found that limiting the real-world distractions increased immersion and subsequently increased memory scores in VR tests. Simulator sickness, on the contrary, is one of the few drawbacks to VR testing, but Lawson (2014) found that an increase in immersion tends to result in decreased simulator sickness.

However, when it comes to actual VR-based tests measuring visual and visuospatial memory related processes, the volume of studies is still scarce. Picard et al. (2017) found that VR-based episodic memory assessment (with a virtual urban environment composed of specific areas, where participants had to memorize as many elements as possible –e.g., scenes, details, spatial, and temporal contexts) was able to show differential recall strategies and patterns associated to increasing age, thus uncovering main developmental differences in feature binding abilities in naturalistic events that are very sensitive to age in comparison with a standard memory assessment. Despite the apparent advantages of VR-based neuropsychological assessment, however, a meta-analysis by Negut et al. (2016) showed that cognitive performance assessed in VR environments is often poorer than performance observed in pen-and-paper or classic computerized testing, leading them to conclude that tasks embedded in VR are more difficult and complex. This leaves open the need to evaluate further the relationship between test performance in VR environments and performance in everyday contexts (i.e., ecological validity –veridicality). Previously, Plancher et al. (2010, 2012) had stated that most episodic memory neuropsychological evaluations are unrelated with events that patients may experience as real memories in their daily lives. This group was the first in using a VR environment to characterize episodic memory profiles in an ecological way (which includes memory for central and perceptual details, spatial–temporal context elements, and fixations) and they concluded that neuropsychological studies would benefit from VR tests and a multitask and multi-component approach in episodic memory evaluation, and that this approach would enhance active information coding in patients suffering from mild to moderate age-associated memory impairment.

More recently, Poos et al. (2021) presented the Object Location Memory Test (OLMT), consisting of a visual perception and memory trial, and the Virtual Tübingen (VT) test, consisting of a scene recognition, route continuation, and route ordering and distance comparison task. Their study showed that such a short digital spatial memory test battery was useful for the detection of object location memory and navigation impairment in patients who ranged from MCI to mild Alzheimer’s Disease dementia.

**Figure 2:**
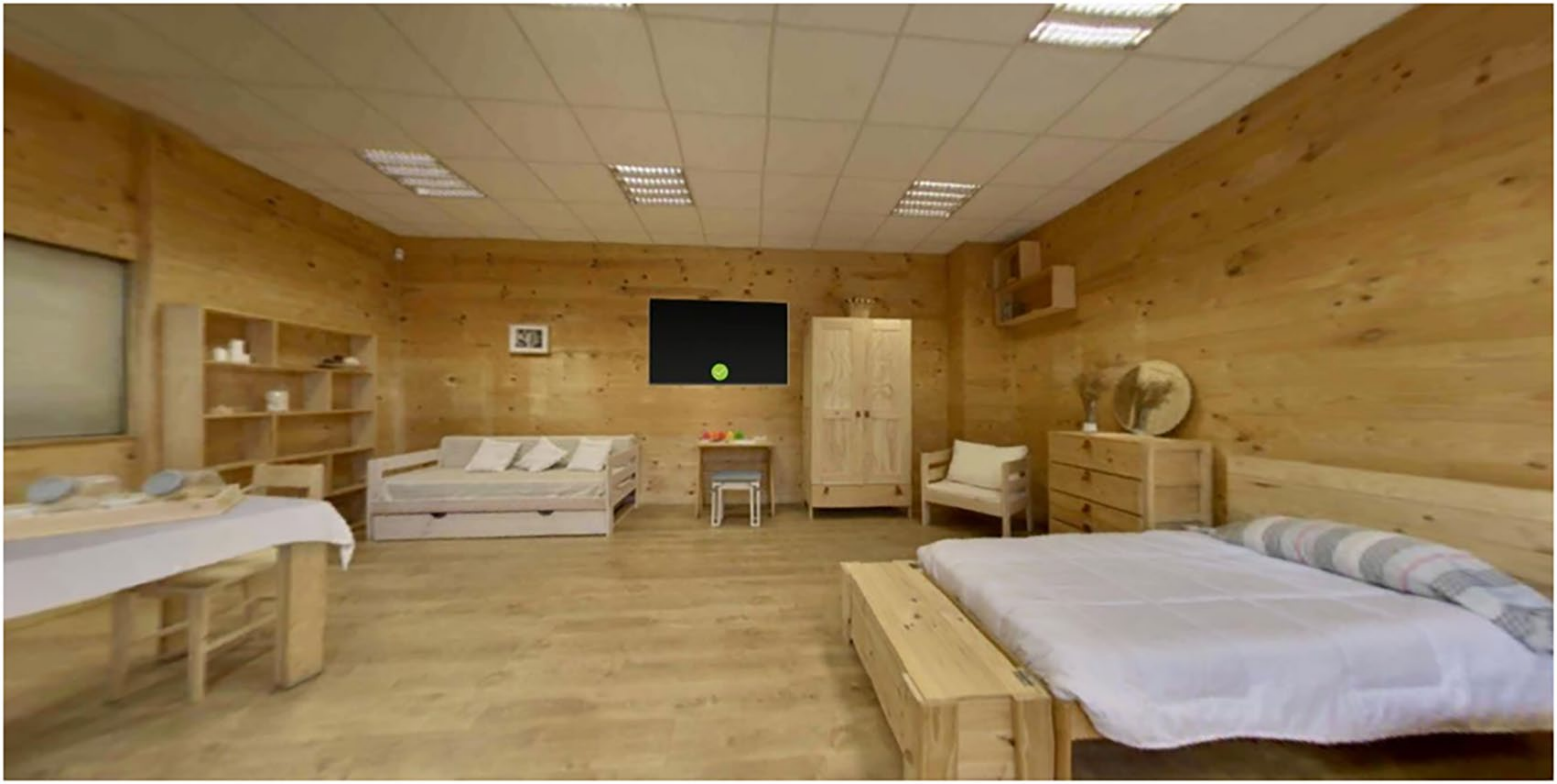
Screenshot of Nesplora Suite test. Reproduced with permission from Giunti-Nesplora Ltd.

Finally, one of the most recent developments in relation to neuropsychological testing of visual memory using Virtual Reality is Nesplora Suite.^1^ The virtual environment is a furniture store, in which the test taker must group different furniture items according to certain conditions so that they are packed and shipped. A voiceover indicates the furniture that you must pack, and the respondent has to point and click on them. They warn us that there are different groups (categories) of people and each one wants lists of between four and six different types of furniture (Figure 2).

In the second task, labelled as source memory task, the user is then shown eight different pieces of furniture or groups of pieces of furniture (e.g., two beds) that have been requested during the previous task, and must decide which group or family have requested them. Only pieces of furniture requested by one single family are presented, and in many cases, they are distinctive elements (e.g., who has asked for two beds? Who has asked for one desk?, etc.). The test reportedly provides measures of the learning curve, immediate and long-term memory, both auditory and visual memory, recognition, prospective memory, primacy and recency effects, and simulation of memory problems. Preliminary access to unpublished data suggests that the source memory task facilitates encoding and storage of items processed in the first visual memory task, and that names given to specific furniture are irrelevant for visual memory performance, which may open the door for the assessment of visual memory in individuals with language problems (i.e., aphasia) that may not rely on verbal cues for the processing of visual items. The updated normative as well as clinical studies for the test are currently in preparation and will show whether this VR-based test can constitute a feasible alternative for a more accurate, ecologically valid neuropsychological assessment of visual memory processes.

## Lessons learned: Present and future lines of neuropsychological assessment of nonverbal memory

There is an obvious need for clinical assessment of episodic memory to be grounded in accepted theoretical models of memory functioning, as well as on solid neuroanatomical models of cognitive function. In other words, memory assessment and detection of memory deficits must reflect our current understanding of memory systems, neuroanatomical models underlying these systems and the clinical reality of how patients present with memory problems. By the time the WMS was replaced by the WMS-in 1987, both our theoretical understanding of memory and our appreciation of the diverse neuroanatomical underpinnings of specific aspects of memory had advanced dramatically and it has advanced even further since then. Memory test development, however, has not always capitalized on these advances—a fact recognized in a number of critiques of test evolution.

Even a cursory look at the clinically available visual memory assessment tools illustrates the multiple challenges that have hampered the development of reliable and valid instruments to assess, in a clinically meaningful way, nonverbal visual episodic memory. With few exceptions, further efforts are required in test development in order to dissociate successfully between visual and verbal memory processes and to assess visual memory more accurately without the confounding influence of verbal mediation and reliance, to a greater or lesser extent, on verbal memory processes. Similarly, with few exceptions, further efforts are required to permit an evaluation of memory for visual detail and memory for spatial locations separately as well as in combination. Furthermore, it is important that clinicians can partial out the impacts of motor and other non-mnestic factors that might influence test performance.

Virtually all neuropsychological tests used in clinical practice are multifaceted. Thus, some or all of the problems with memory tests detailed above in relation to assessment of visual memory are not unique to this specific domain. The brain structures and networks that mediate memory functioning are complex and diverse, and the tests used in clinical practice should be specific in identifying the nature and causes of observed problems. The challenge for test developers and clinicians is to determine ways in which we can recognize and then partial out the contributions of distinct cognitive (and noncognitive) factors that impact performance, which would inevitably vary depending on the specific nature of the visual material used (e.g., faces, abstract designs, or ecologically meaningful objects) and the test conditions (e.g., requirement for visual/visuo-spatial learning over repeated trials and free recall vs. recognition). The challenge is also to map specific components of the tasks to specific neuroanatomical regions.

Overall, an accurate assessment of visual memory and visual–spatial memory processes needs to rely on tests that minimize the confound of visual and visual–spatial memory with other cognitive domains. Failure to acknowledge the multifaceted nature of virtually any visual and/or visual–spatial memory instrument, some of which may lure clinicians to create composite scores of what are dissociable cognitive skills, is likely to mislead the clinical interpretation of test performance, obscuring the brain-behavior specificity that exists in relation to discrete cognitive processes recruited by the instrument, potentially resulting in a misled diagnostic process and/or relevant intervention approach with the targeted patients. This is particularly important in neurodegenerative conditions in which the incipient cognitive deficits are often subtle and confined to relatively isolated cognitive domains associated with distinct neuroanatomical regions. The identification of these early symptoms is crucial for the early diagnosis of the suspected underlying pathophysiological processes. Indeed, an early and accurate description of preserved and defective cognitive processes is at the core of neuropsychological rehabilitation plans.

As clinicians, we must always appreciate the complexities of our assessment tools (what they actually measure rather than what they are purported to measure), particularly if we are to draw inferences about the neuroanatomical underpinnings of detected deficits. The challenge for researchers and clinicians is to develop nonverbal analogues of some of the best verbal memory test instruments that might then be capable of capturing, either as a stand-alone test or as part of a wider nonverbal memory test battery, multiple aspects of nonverbal memory (e.g., impacts of learning trials and of interference trials, short and long delayed free and cued recall, and recognition memory), but also, in line with the growing appreciation of the importance of a process-based approach to neuropsychological assessment, qualitative aspects of the individual’s performance (learning strategies and error types) that might facilitate identification of reasons or potential reasons for “memory” failure. However, in adopting such an approach, it is important to bear in mind the wisdom of those who have advised us that simply developing a nonverbal analogue of a verbal memory test will not ensure that it will tap solely the right-hemisphere contributions to memory. We should, instead, be guided by the clinical and research community and we should seek first to identify those tests that have demonstrated laterality effects in temporal lobe epilepsy. Such tests might then form the basis of new memory tests capable of characterizing and identifying laterality effects in memory, where they exist, as well as within hemisphere differences in memory structure and function. Such an approach would go some way to addressing what Saling (2009) identified as the problem with adopting a strict material-specificity principle that sees clear separation of function between the hemispheres. Until such tests have been developed and are widely available to clinicians, caution is advised when seeking to interpret test results with our current tools.

To paraphrase Hoelzle et al. (2011), test development must be grounded in a clear empirical framework. Only then can the psychometric properties of the test, as well as its clinical utility, be examined fully. As clinicians, however, we must not lose sight of the complexity of even the simplest of cognitive tasks and we must be prepared to accept the challenges of undertaking a comprehensive neuropsychological assessment as opposed to neuropsychological testing alone.

Finally, in relation to computerized tests and the ongoing development of VR-based neuropsychological tests, recent developments may prove to be an asset for clinical neuropsychological assessment, but these new developments must demonstrate that they can overcome the barriers to reliable and valid assessment presented throughout this review. While the problems posed by use of abstract stimuli and the interference of graphomotor processes seem easily solved by means of currently existing VR-based evaluation paradigms, there are still open questions about the other two main challenges. On the one hand, it will take further research to establish whether existing VR-based neuropsychological tests can ensure the assessment of visual and visuospatial processes with a minimum or no verbal mediation. On the other hand, to the best of our knowledge, there are no specific computerized or VR-based neuropsychological tests that have been designed specifically to shed light on inter and intra-hemispheric lesions. As Saling (2009) reminded us, individual differences in performance within different domains reflects the fact that neither verbal nor non-verbal memory should be considered a unitary construct. No one test can be expected to provide a comprehensive assessment of either. Furthermore, different strategies can be adopted to facilitate learning and memory of both verbal and nonverbal material—adding complexity to test interpretation. While obviously presenting a challenge for test developers, this fractionation of memory is especially relevant for an accurate examination of dissociations between visual and spatial memory processes, for a clearer identification and understanding of deficits and their underlying causes, and for a more individually tailored plan for neuropsychological rehabilitation interventions in clinical settings.

In other words, from a cost–benefit analysis point of view, and for clinicians to adopt costly new technology and VR based neuropsychological tests, these tools need to address the challenges posed by more than 50 years of imperfect visual and visuospatial memory assessment paradigms.

## Author contributions

All authors formulated the research question(s). UD-O and BR performed the literature review and wrote the initial draft of the paper. AB-C and TB collaborated in writing and developing the review further. UD-O, AB-C, and TB contributed with additional literature and critical reviews and comments of the working draft. TB performed final edits. All authors contributed to the article and approved the submitted version.

## Funding

This research was funded with authors’ own resources.

## Conflict of interest

The authors declare that the research was conducted in the absence of any commercial or financial relationships that could be construed as a potential conflict of interest.

## Publisher’s note

All claims expressed in this article are solely those of the authors and do not necessarily represent those of their affiliated organizations, or those of the publisher, the editors and the reviewers. Any product that may be evaluated in this article, or claim that may be made by its manufacturer, is not guaranteed or endorsed by the publisher.

## Footnotes

^1^ https://nesplora.com/en/producto/nesplora-memory-suite-2/
